# EZH2 Inhibition Compromises α4-1BB-Mediated Antitumor Efficacy by Reducing the Survival and Effector Programming of CD8^+^ T Cells

**DOI:** 10.3389/fimmu.2021.770080

**Published:** 2021-11-24

**Authors:** Christopher J. Stairiker, Sophia Xiao Pfister, Eleanore Hendrickson, Wenjing Yang, Tao Xie, Catherine Lee, Haikuo Zhang, Christopher Dillon, Graham D. Thomas, Shahram Salek-Ardakani

**Affiliations:** ^1^ Cancer Immunology Discovery, Worldwide Research, Development Medical, Pfizer Inc., San Diego, CA, United States; ^2^ Translational Sciences, Worldwide Research, Development Medical, Pfizer Inc., San Diego, CA, United States; ^3^ Computational Biology, Worldwide Research, Development Medical, Pfizer Inc., San Diego, CA, United States

**Keywords:** EZH2, CD8, T cell, Bim, CD137 (4-1BB)

## Abstract

Enhancer of Zeste Homolog 2 (EZH2) inhibitors (EZH2i) are approved to treat certain cancer types. Previous studies have suggested the potential to combine EZH2i with immune checkpoint blockade targeting coinhibitory receptors like PD-(L)1 and CTLA-4, but whether it can also enhance the activity of agents targeting costimulatory receptors is not known. Here, we explore the combination between EZH2i and an agonist antibody targeting the T cell costimulatory receptor 4-1BB (α4-1BB). Our data show that EZH2i compromise the efficacy of α4-1BB in both CT26 colon carcinoma and in an *in vivo* protein immunization model. We link this to reduced effector survival and increased BIM expression in CD8^+^ T cells upon EZH2i treatment. These data support the requirement of EZH2 function in 4-1BB-mediated CD8^+^ T cell expansion and effector programming and emphasize the consideration that must be given when combining such antitumoral therapies.

## Introduction

Currently, EZH2 (Enhancer of Zeste Homolog 2) inhibitors (EZH2i) are being tested in the clinic in multiple cancer indications, and this targeted cancer therapy is approved for use in epithelioid sarcoma and relapsed/refractory follicular lymphoma (1, 2). Among the EZH2i being evaluated in the clinic is PF-06821497, a SAM competitive inhibitor in Phase I trials (3, 4). Many cancer indications overexpress EZH2, including prostate, breast, and bladder, and gain-of-function mutations suggest that cancer cells utilize this pathway to promote tumor progression (5). EZH2 participates in the chromatin-modifying Polycomb Repressive Complex 2 (PRC2), where it catalyzes di- and trimethylation of histone 3 lysine 27 (H3K27) residues to silence target gene expression (6). EZH2 is primarily expressed in proliferating cells, establishing the epigenetic landscape on newly synthesized histones (6). In murine models, EZH2i have demonstrated combinatorial potential with checkpoint blockade immunotherapy (CBI), such as αPD-1 in prostate (7), head and neck (8), and bladder cancers (9) as well as αCTLA-4 in melanoma (9, 10). The efficacy is attributed to effects on cancer cells as EZH2i induce expression of antigen presentation-associated genes (8, 10) and chemokine ligands (9, 10). Furthermore, EZH2i are reported to affect immune cells, notably by decreasing Treg suppressive capacity (9). These mechanisms all enhance the recruitment and activation of the immune system, particularly CD8^+^ T cells (9, 11, 12). Although these studies would suggest that EZH2 may be dispensable for T cell function, models of graft versus host (13, 14), acute viral infection (15, 16), and melanoma (17), would suggest a T cell-intrinsic requirement for effector cells.

Previous studies have demonstrated the potential for EZH2i to synergize with αPD1 and αCTLA-4 CBI that enhance anti-tumor immunity by derepressing TCR-mediated signaling (18) and impairing Treg function (11), respectively. However, little is known about the possibility of combining EZH2i with alternative immunotherapeutic approaches that target CD8^+^ T cells (9, 10). CD137, or 4-1BB, is a costimulatory molecule expressed on recently activated T cells. 4-1BB agonism can potentiate CD8^+^ T cell cytotoxicity, increase proliferation, and promote survival, ultimately contributing to enhanced antitumor immunity (19–21). Clinically, 4-1BB agonists are being evaluated and have demonstrated efficacy in human *ex vivo* culture systems linked to increased CD8^+^ responses (22, 23). This success has led to combination strategies of α4-1BB with other therapies to augment tumor regression, but the combination of EZH2i has yet to be explored (22).

Here, we test the combination therapy between EZH2i and an agonistic α4-1BB antibody. We show that EZH2i compromise the efficacy of α4-1BB, limiting the CD8^+^ T cell response. Using *in vivo* and *in vitro* models, we confirm the impact EZH2i have on CD8^+^ T cells in terms of survival and function. These data highlight the importance of exploring the impact of targeted chemotherapy on immune-intrinsic biology and further highlight the judicious consideration of combination partners when considering therapeutic combinatorial approaches.

## Materials and Methods

### Animals

BALB/c and C57BL/6 female mice were housed in a specific pathogen-free vivarium at Pfizer Inc (San Francisco or San Diego, CA) or Crown Bioscience Inc. (San Diego, CA). All experiments and procedures were conducted under approved protocols by the Institutional Animal Care and Use Committee (IACUC) (LAJ-2019-01347).

### Tumor Experiments

For CT26 tumor inoculations, 0.6 x 10^6^ CT26 tumor cells were subcutaneously injected into BALB/c mice. Six days post-implantation, mice received either vehicle, 30 mg/kg (mpk), or 100 mpk of the EZH2 inhibitor PF-06821497, daily by subcutaneous injection. Mice received 3 doses of 10 mpk mouse IgG1 isotype control (BioXcell, BE0083) or α4-1BB antibody (mAb9371, inhouse generated).

For MC38 tumor inoculations, 0.25 x 10^6^ MC38 tumor cells were injected into C57BL/6 mice and treated daily with 100 mpk PF-06821497 or vehicle control from implantation. On days 9, 12, and 15, mice were given either isotype control or α4-1BB at 3 mpk.

### Protein Immunization Experiments

Naïve CD45.2^+^ OT-I CD8^+^ T cells were isolated *via* the EasySep Mouse Naive CD8^+^ T cell Isolation Kit (STEMCELL, 19858). Cells were adoptively transferred intravenously on day -1. On day 0, mice were subcutaneously injected with 100 μg of ovalbumin (Endofit, Vac-pova) and 50 μg of Poly I:C (Tocris, 4287). Starting on day 0, mice also received subcutaneous injections of vehicle, 30 mpk or 100 mpk PF-06821497 daily for a total of 10 days. On day 1, mice received a single intraperitoneal injection of 5 mpk IgG1 isotype control or α4-1BB. At the study endpoint, blood and spleen were aseptically removed for processing.

### Tissue Processing

Tissues were processed as previously described (24). In brief, tumors were dissociated with the Tumor Dissociation Kit (Miltenyi, 130-096-730). For dissociated spleens and blood, red blood cells were lysed *via* RBC Lysis Buffer (eBioscience, 00-4333-57). Spleens and tumors were enumerated using a Beckman ViCell XR Automated Cell Viability Analyzer. For *ex vivo* stimulations, cells were treated as described previously (25).

### *In Vitro* CD8^+^ T Cell Activation

Murine CD8^+^ T cells were isolated (STEMCELL, 19853) and activated with 1 μg/mL αCD3 (eBioscience, 16-0031-86) and 2.5 μg/mL αCD28 (eBioscience,16-0281-82) for 2 days prior to subculture in the presence of 20 U/mL recombinant murine IL-2 (Peprotech, 50-813-288).

For human T cell culture, frozen negatively isolated CD8^+^ T cells (Hemacare, PB08NC1) were activated with CD3-CD28 Dynabeads (Gibco, 11131D) for 72 hours in the presence of 20 U/mL IL-2 (Roche, 11011456001) and 5 ng/mL IL-7 (Peprotech, 200-07) and IL-15 (Peprotech, 200-15). Cells were subcultured with fresh media and cytokines.

### Flow Cytometry

Cells were stained as previously described (25). In brief, cells were stained for surface antigens (**Table S1**), permeabilized with the FoxP3/Transcription Factor permeabilization Kit (eBioscience, 50-112-9060) and stained for intracellular targets (**Table S1**) before fixation with 1% formaldehyde (RICCA, 3180-16), and acquired on an BD LSRFortessa, or Cytek Aurora. Post analysis was performed using FlowJo v10 Software. Statistical analysis and data representations were generated using GraphPad Prism version 9.

### scRNA-Sequencing

For MC38 tumor inoculations, 0.25 x 10^6^ MC38 tumor cells were subcutaneously injected into C57BL/6 mice on day 0. One or ten days after implantation mice received 100 mpk dosing of PF-06821497 or vehicle control, subcutaneous, daily. CD45^+^ cells were isolated using CD45 TIL microbeads (Miltenyi, 130-110-618) and libraries were generated using the Single Cell 3’ GEM, Library and Gel Bead Kit v3 (10X Genomics, PN-1000075), Chromium Single Cell B Chip (PN-100073), and Chromium i7 Multiplex Kit (PN-120262). Sequencing was performed by Novogene and downstream analysis performed using Seurat. Initial processing of scRNA-Seq data was performed using Cell Ranger (10X Genomics) and then further analyzed using Seurat. A cut-off of greater than 200 genes and less than 5000 genes per cell was used to filter samples. Cells with UMI greater than 10% that were attributable to mitochondrial genes were excluded. Genes were only analyzed if they were present in a minimum of three cells. Normalizing and scaling was performed using the default settings in Seurat. A total of 29559 (day 7 vehicle), 71848 (day 7 EZH2i), 23404 (day 16 vehicle), and 13757 (day 16 EZH2i) were pooled and used for analysis after initial QC. Subsequent reclustering of lymphoid cells was performed.

## Results

### EZH2i Compromises α4-1BB Efficacy

**Figure 1:**
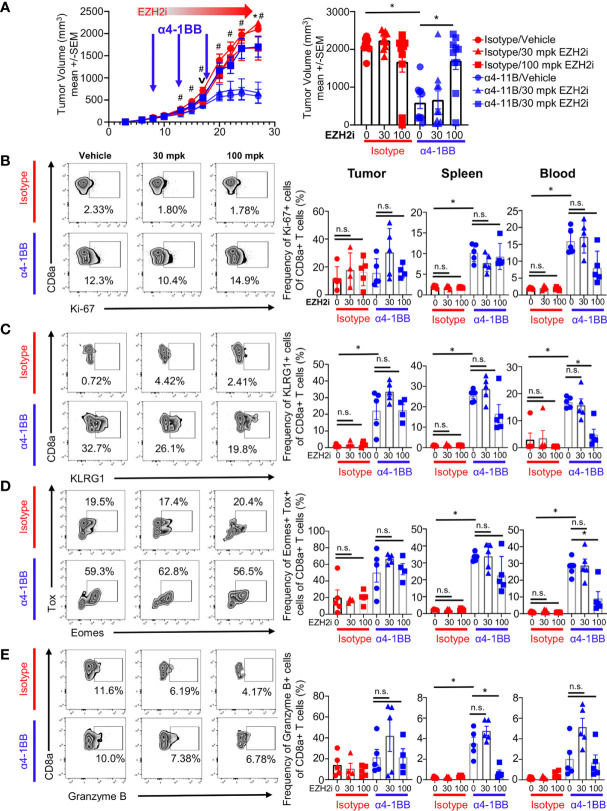
EZH2i compromise α4-1BB efficacy. CT26-tumor bearing mice were treated daily with a single s.c. injection of EZH2 inhibitor starting on day 6 post implantation and then treated with α4-1BB starting on day 8, every 5 days for a total of three doses. Tumor growth curves (**A**, left panel) and final tumor volumes (**A**, right panel) are shown for treatment groups. Mice harvested at day 17 (v) for T cell phenotypes were analyzed by flow cytometry. Representative FACs plots from tumor samples (**B–E**, left panel) and pooled data dot plots from tumor, spleen, and blood (**B–E**, right panel) are shown for proliferating CD8^+^ T cells **(B)**, activated effectors **(C, D)**, and cytotoxic cells **(E)**. Mice treated with isotype are marked in red; mice treated with α4-1BB are marked in blue. Vehicle treated control mice are marked with circles; triangles indicate 30 mpk EZH2i treatment while squares indicated 100 mpk EZH2i treatment. For A, asterisks (*) indicate p<0.05 as determined by ANCOVA for comparison of groups treated with α4-1BB with and without 100 mpk dosing with EZH2i while hashtags (#) indicate p<0.05 for comparison of groups treated with vehicle control with and without α4-1BB. For B-E, Asterisks (*) indicate p<0.05 as determined by 2way ANOVA and *post hoc* comparison of group means. n.s. indicates non-significant result. n≥ 9 mice per group for tumor growth inhibition studies **(A)**. n≥ 4 mice per group for T cell analysis **(B–E)**.

To characterize the effects of EZH2i on T cells, we utilized the CT26 colorectal cancer model in combination with α4-1BB therapy, which demonstrates robust single-agent efficacy in this system (26). We began daily dosing of tumor-bearing mice with the EZH2 inhibitor PF-06821497, starting on day 6 post-implantation (**Figure 1A**) (3). Two days later, the 4-1BB agonist antibody mAb9371 was administered to induce the proliferation and expansion of recently activated CD8^+^ T cells (26). The α4-1BB treatment and vehicle control combination resulted in robust tumor growth control, whereas vehicle control and isotype treatment led to tumor outgrowth (**Figure 1A**). Surprisingly, rather than enhancing, high dose EZH2i completely abrogated the efficacy of α4-1BB therapy (**Figure 1A**). To understand how EZH2i mediated this effect, we analyzed tumors, spleens, and blood from mice at day 17 post-implantation. This time point was chosen as immediately proximal to the deviation in tumor volumes elicited by α4-1BB treatment. Therefore, it should allow us to identify changes in immune profiles that result from treatment conditions and not differences in tumor volumes. We observed no consistent differences in total CD4^+^ or CD8^+^ T cell frequencies between treatment groups in any of the tissues assessed (**Figure S1A**). Previous literature has suggested that Tregs rely on EZH2 for suppressive capacity (27). However, we did not observe any impact on the frequency of this population (CD4^+^Foxp3^+^) upon EZH2i treatment (**Figure S1B**). While there were no differences in frequency, we did observe differences in the phenotype of CD8^+^ T cells upon EZH2i treatment. Total proliferating cells marked by Ki-67 expression were relatively equal (**Figure 1B**); however, the increase in activated KLRG1^+^ CD8^+^ T cells mediated by α4-1BB was reduced at 100 mg/kg (mpk) EZH2i, particularly in the blood (**Figure 1C**). Further supporting a defect in the effector CD8^+^ T cell program, the α41-BB mediated increase in CD8^+^Eomes^+^TOX^+^ committed effectors was significantly lower in the blood and somewhat depleted in the spleen at 100 mpk EZH2i (**Figure 1D**). Furthermore, this high dose of EZH2i also reduced Granzyme B expressing CD8^+^ T cells in the spleen (**Figure 1E**).

This loss of α4-1BB efficacy by EZH2i was not only observed in the CT26 model but also using the murine adenocarcinoma C57BL/6-based MC38 model (**Figure S1C**). Altogether, these data show that EZH2i at high concentrations can compromise the tumor growth control mediated by agonistic α4-1BB therapy.

### EZH2i Reduce Survival and Expansion of Responding CD8^+^ T Cells

**Figure 2:**
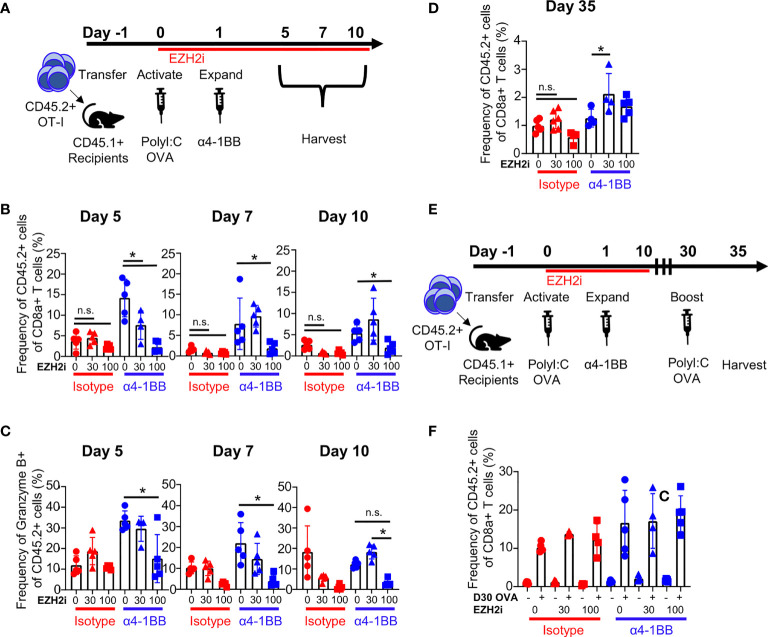
EZH2i reduce the magnitude of the antigen-specific effector response. Recipient mice (CD45.1^+^) received congenically mismatched donor (CD45.2^+^) OT-I^+^ cells before being activated with OVA and PolyI:C, dosed with EZH2i and α4-1BB **(A)**. On days 5, 7, and 10, the frequency of donor OT-I^+^ cells **(B)** and donor cells producing Granzyme B **(C)** was determined in isotype-treated (upper panel) and α4-1BB-treated (lower panel). On day 35 post activation, frequency of donor OT-I^+^ cells was detected in recipient animals **(D)**. On day 30, one cohort of mice was given a second injection of OVA and PolyI:C and both cohorts (untreated memory and OVA/PolyI:C-treated memory) were taken down 5 days later **(E)** and relative expansion compared to an untreated cohort was determined **(F)**. Mice treated with isotype are marked in red; mice treated with α4-1BB are marked in blue. Vehicle treated control mice are marked with circles; triangles indicate 30 mpk EZH2i treatment while squares indicated 100 mpk EZH2i treatment. Asterisks (*) indicate p<0.05 as determined by 2way ANOVA and *post hoc* comparison of group means. n.s. indicates non-significant result. n≥ 4 mice per group for immunization studies **(A–C)**. n≥ 3 mice per group for memory studies **(D–F)**.

Our tumor data show that EZH2i influences CD8^+^ T cell effector functions. However, multiple factors within the TME could explain the loss of efficacy when EZH2i were combined with α4-1BB. To directly determine the impact of EZH2i on the CD8^+^ T cell response *in vivo*, we utilized a protein immunization model. We took advantage of the OT-I system, in which CD8^+^ T cells recognize the immunodominant epitope of the ovalbumin (OVA_257-264_, SIINFEKL), to understand the effects of EZH2i on the antigen-specific T cell response. We performed an adoptive transfer of naïve OT-I^+^ cells into congenically mismatched recipient mice and subsequently immunized these mice with ovalbumin and TLR3 agonist Poly I:C at which point we began dosing them with EZH2i. One day later, a single dose of α4-1BB was administered to expand early activated cells. We then performed a kinetic study to characterize the CD8^+^ effector response (**Figure 2A**). By staining for the H3K27 trimethylation (H3K27me3) status, we confirmed the efficacy of EZH2i on donor OT-I cells (**Figure S2A**). As expected, α4-1BB treatment increased the expansion of donor OT-I cells compared to isotype-treated mice; however, treatment with high dose EZH2i significantly reduced the recovery of donor cells on days 5 and 7 post-activation (**Figure 2B**). Consistent with high dose EZH2i impacting effector T cells, we observed the lowest recovery of donor cells in the isotype-treated control mice receiving 100 mpk EZH2i across all time points (**Figure 2B**). Treatment with α4-1BB did not lead to a significant increase in EZH2 expression compared to isotype-treated mice. However, among mice treated with α4-1BB, the combination with 100 mpk EZH2i led to a significant decrease in EZH2 expression (**Figure S2B**). Despite attempts to determine the effects of EZH2i on donor OT-I cells at day 3 post-activation, we were unable to detect a reliable donor population for analysis (**Figure S2C**). These differences in OT-I frequencies were also consistent with the absolute number of OT-I donor cells recovered from these mice (**Figure S2D**).

During acute viral infection, KLRG1^hi^CD127^lo^ cells are considered short-lived effector cells (SLEC), while KLRG1^hi^CD127^lo^ cells are classified as memory precursor effector cells (MPEC) (28). Using this paradigm, high dose EZH2i increased KLRG1^hi^CD127^lo^ frequencies following α4-1BB treatment on days 5 and 7 (**Figure S2E**). As KLRG1^hi^CD127^lo^ cells have enhanced effector capacity, in theory, these cells should be more cytotoxic (29). However, in agreement with our tumor model data (**Figure 1E**), we observed that the frequency of Granzyme B^+^ OT-I was considerably lower in both the α4-1BB and isotype-control treated groups treated with high-dose EZH2i (**Figure 2C**). This reduction in Granzyme B positivity was specific to the CD8^+^ donor T cells, as Granzyme B expression in NK cells was not affected by EZH2i (**Figure S3A**). This effect was also specific to cytotoxic capacity as restimulation with cognate antigen *ex vivo* did not influence the frequency of TNFα^+^IFNγ^+^ polyfunctional effector cells (**Figure S3B**). These findings are consistent with reports that ablation of *Ezh2* in CD8^+^ T cells during acute infection only mildly affected cytokine production despite differences observed in relative frequencies of KLRG1^hi^CD127^lo^/CD127^hi^KLRG1^lo^ populations (15).

To investigate the potential effects of EZH2i on skewing CD8^+^ memory responses, we evaluated OT-I frequencies and phenotypes at day 35 post-immunization. Only a modest reduction in the frequency of donor cells at a day 35 timepoint was observed in the 100 mpk EZH2i isotype control treatment group. Interestingly, in groups treated with α4-1BB and 30 mpk EZH2i, there was a significant increase in the recovery of donor cells compared to α4-1BB treatment alone (**Figure 2D**). To determine whether brief exposure to EZH2i impacts the memory population, we performed a protein restimulation assay to test whether EZH2i leads to a functional enhancement of CD8^+^ memory responses. Mice were immunized with OVA protein as before and given a second dose of OVA on day 30 (**Figure 2E**). To assess recall responses, we measured the expansion of the memory compartment five days later (**Figure 2F**). Despite the mild reduction in donor cells with high dose EZH2i, this group proliferated to the same extent as control and low dose treatment groups (**Figure 2F**). It also possessed equivalent cytotoxic molecule expression (**Figure S3C**). From these data, we can conclude that high dosing with EZH2i compromises α4-1BB-mediated effects on effector CD8^+^ T cells but does not affect memory formation and recall responses.

### Loss of Survival and Cytotoxic Programming With EZH2i

**Figure 3:**
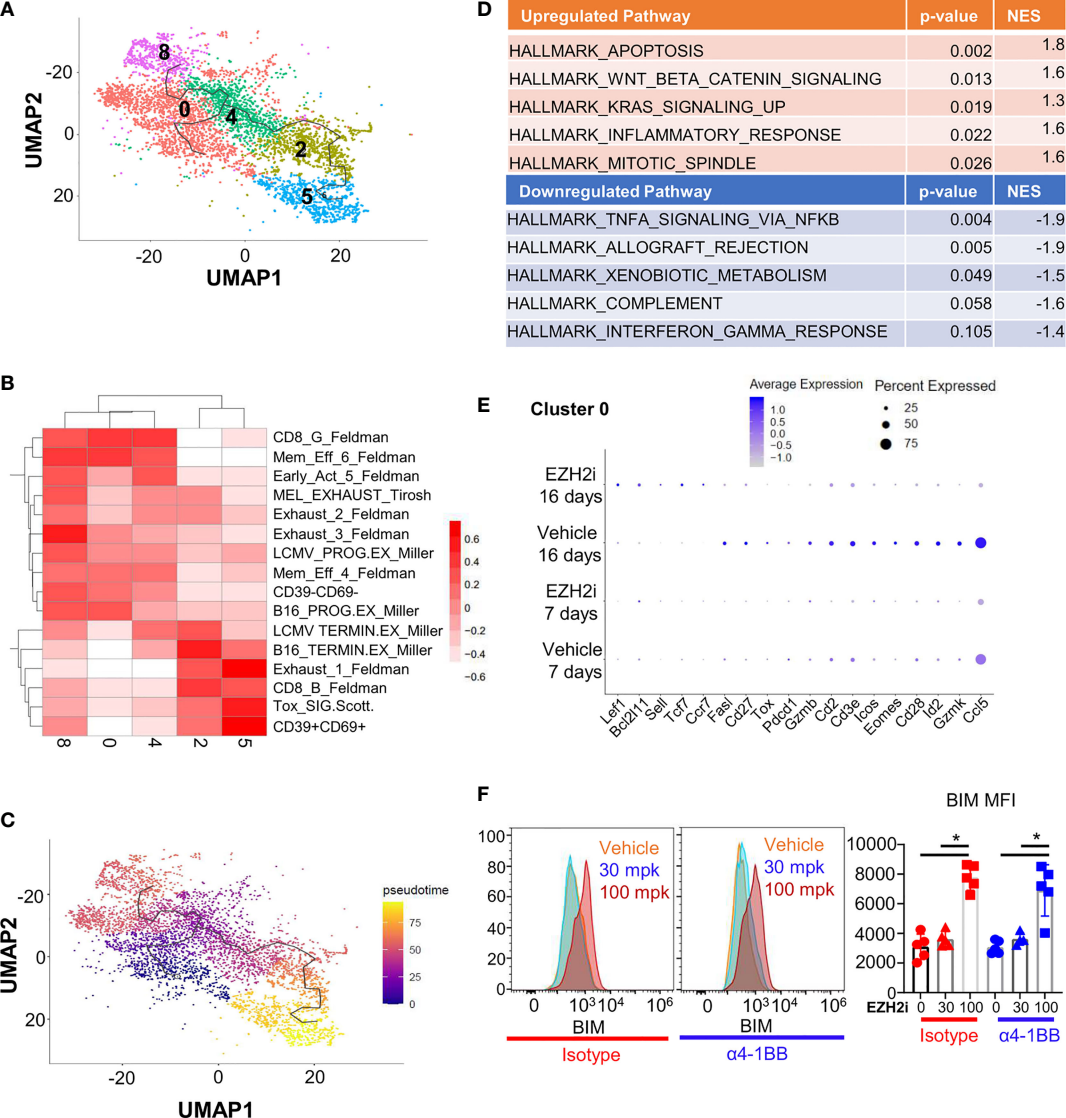
EZH2i impact the transcriptional effector program and pro-apoptotic protein expression. MC38 tumor cells were implanted in naïve mice and treated for 16 or 7 days prior to takedown with 100 mpk EZH2i. CD45^+^ T cells were isolated and scRNA-Seq performed with subsequent lymphocyte reclustering and focusing on CD8^+^ clusters **(A)**. GSVA heatmap depicting CD8^+^ clusters aligned to publicly available CD8^+^ populations found in exhaustion models **(B)**. Monocle analysis was performed to determined trajectory of differentiation within CD8^+^ clusters in pseudotime **(C)**. Pathways most impacted in CD8^+^ T cells by EZH2i treatment were determined by comparing DEG lists to Hallmark molecular signatures database. The top 5 up- and downregulated pathways are shown **(D)** and the complete lists can be found in **Tables S2**, **S3**. Dot plot of curated DEG of interest from the cluster with the most DEG (Cluster 0) are depicted **(E)**. BIM levels were determined *in vivo* at day 5 post OVA immunization (from **Figure 2**) **(F)**. Representative histograms (**F**, left two panels) and pooled data (**F**, right) were determined by intracellular flow cytometry. Mice treated with isotype are marked in red; mice treated with α4-1BB are marked in blue. Vehicle treated control mice are marked with circles; triangles indicate 30 mpk EZH2i treatment while squares indicate 100 mpk EZH2i treatment. NES indicates normalized enrichment score. Asterisks (*) indicate p<0.05 as determined by 2way ANOVA and *post hoc* comparison of group means.

The deposition of repressive H3K27me3 residues can inhibit the expression of many genes, potentially influencing multiple cellular programs. To understand how EZH2i affects antitumoral CD8^+^ T cells, we performed scRNA-Seq profiling of tumor-infiltrating immune cells. CD45^+^ cells were isolated on day 17 post-implantation from MC38 tumor-bearing mice that had either been treated with EZH2i for 16 days or 7 days before isolation (**Figure S4A**). Clustering of tumor-infiltrating lymphocytes identified 12 total clusters (**Figure S4B**), of which 5 were determined to be CD8^+^ T cells (**Figure S4C**). To determine the phenotype of these five clusters (Clusters 0, 2, 5, 4, and 8) (**Figure 3A**), we compared the gene signatures of each intratumoral CD8^+^ T cell population against signatures of CD8^+^ T cell activation and exhaustion obtained from public datasets (30) using gene set variation analysis (GVSA) (**Figure 3B**) and queried genes characteristic of tumor-infiltrating T cell phenotypes (**Figure S4D**). Our results identified the expected distribution of intratumoral CD8^+^ T cell phenotypes, including stem-like (Clusters 0,4, and 8), effector-like (Cluster 2), and more terminally exhausted (Cluster 5) clusters (**Figure 3B**). Pseudotime trajectories generated by Monocle support a transitional relationship between these stem-like and effector/exhausted fates (**Figure 3C**). We assessed the relative fraction of each CD8^+^ T cell cluster across groups in our dataset; however, we did not observe significant differences, consistent with our earlier observations that tumor CD8^+^ T cell populations are not dramatically changed in the TME at day 17 (**Figure S4E**). Considering that the effects of EZH2i will likely be more pronounced after 16 days of treatment, we queried the gene pathways and cellular functions that were impacted by inhibition of EZH2 after 16 days of treatment *in vivo*. We compared the effects of EZH2i to vehicle control across all CD8^+^ T cell subsets using the Hallmark Gene Set Collection, observing that the top pathways affected by EZH2i treatment were effector and memory gene programs as well as apoptosis (**Figure 3D**) (**Tables S2, S3**). To identify molecular mechanisms associated with CD8^+^ T cell dysfunction upon EZH2i treatment, we assessed changes in gene expression within individual CD8^+^ T cell clusters. Cluster 0 was found to be the most impacted as evidenced by over 500 differentially expressed genes (DEG) compared to the other clusters, Cluster 2 (88 DEG), Cluster 4 (0 DEG), Cluster 5 (51 DEG), and Cluster 8 (17 DEG). Cluster 0 was found to possess an early activation gene signature (**Figure 3B**), impaired T cell activation-associated gene pathways (**Figure 3D**), and the most extensive changes in gene expression. Thus, EZH2i impair the early effector differentiation of CD8^+^ T cells, consistent with our tumor and OVA immunization results.

Focusing on Cluster 0, EZH2i significantly increased memory-associated genes, including *Tcf7* (encoding TCF-1), *Lef1* (LEF-1), *Ccr7* (CCR7), and *Sell* (CD62L) after 16 days of treatment, and a similar trend was observed after only 7 days (**Figure 3E**). Concordantly, other clusters after both 7 and 16 days of treatment expressed a similar pattern (**Figure S5A**). Our protein immunization experiments supported these findings, where 100 mpk dosing with EZH2i resulted in increased TCF-1 levels (**Figure S5B**) and more central memory (CD44^+^CD62L^+^) phenotype (**Figure S5C**). These characteristics demonstrate that inhibiting EZH2 during the effector phase can have long-term effects on the memory population, as dosing with EZH2i was discontinued after day 10 post-immunization.

Consistent with reduced effector pathway activation, treatment with EZH2i was associated with a reduction in effector-associated molecules in Cluster 0, including *Id2* (ID2), *Gzmb* (Granzyme B), *Gzmk* (Granzyme K), *Ccl5* (CCL5/RANTES), and *Tox* (TOX) (**Figure 3E**). Notably, we observed increased expression of *Bcl2l11* encoding pro-apoptotic protein BIM was significantly increased in this cluster (**Figure 3E**) and more highly expressed in other clusters with EZH2i treatment (**Figure S5A**). Previous work targeting EZH2 in T cells purported that loss of BCL2 was responsible for the survival defect in activated CD8^+^ T cells, but *Bcl2* was not identified as being differentially expressed in this cluster (31). However, as the balance of these apoptotic molecules can impact CD8^+^ T cell effector survival, this increased pro-apoptotic BIM expression could explain the reduced survival of effector cells *in vivo* (32). Using our previous immunization model (**Figure 2A**), we determined the BIM levels in early effectors at day 5 post OVA immunization. We discovered a significant increase in BIM levels with 100 mpk dosing of EZH2i (**Figure 3F**). BCL2 levels are also significantly increased, although not as much as BIM (**Figure S6A**). This relative fold induction of BIM over BCL2 decreased over time, restoring the balance between pro- and anti-apoptotic molecules that could explain the intact memory pool (**Figure S6B**). This scRNA-Seq data suggested that EZH2i influences the stability of recently activated CD8^+^ T cells. Considering the reduced recovery of effector cells post treatment, our data suggest that viability may be influenced *via* increased pro-apoptotic BIM expression and imbalanced memory- and effector-associated gene profiles.

### EZH2i Compromises Activated CD8^+^ T Cell Viability *In Vitro*

**Figure 4:**
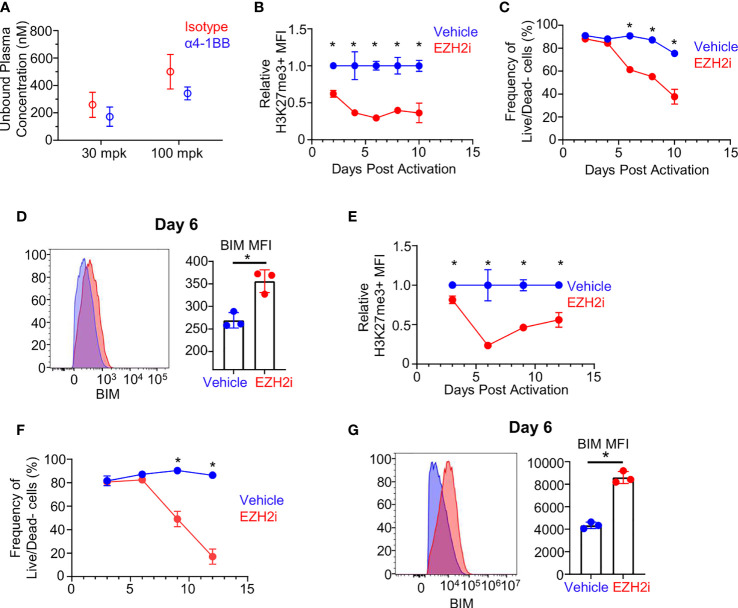
Effect of EZH2i on BIM expression and effector survival is CD8^+^ T cell intrinsic. Biologically relevant exposure of *in vivo* activated CD8^+^ T cells was determined by calculating the free unbound plasma concentration of EZH2i **(A)** (from mice in **Figure 2**) at 3 hours after treatment on day six post OVA/PolyI:C administration. Using the 100 mpk exposure concentration (~500 nM), isolated and activated murine CD8^+^ T cells were exposed to EZH2i and the relative reduction in H3K27me3 status **(B)** and viability **(C)** were tracked over time. BIM levels were determined on day 6 post activation. Representative histogram (**D**, left panel) and pooled data (**D**, right panel) are shown. Isolated human CD8^+^ T cells were activated *in vitro* and relative reduction of H3K27me3 status **(E)** and viability **(F)** were determined. Human BIM levels were determined at day 6 post activation. Representative histogram (**G**, left panel) and pooled data (**G**, right panel) are shown. Blue indicates vehicle-treated cultures. Red indicates EZH2i treated-cultures at 500 nM (mouse) or 300 nM (human) concentrations. Gaussian distribution was determined by normality test and subsequent appropriate statistical test, unpaired student’s t-test or Mann-Whitney U, was performed to determine significance. Asterisks (*) indicated significant findings; p ≤ 0.05. For **(A–D)**, results representative of 2 independent experiments.

The loss of donor recovery with high dose EZH2i could be explained by skewed differentiation, reduced survival, or a combination of these two factors. To gain more insight into cell-intrinsic effects of high dose EZH2i on effectors, we exposed isolated murine CD8^+^ T cells to biologically relevant concentrations of EZH2i *in vitro* (**Figure 4A**). Confirming the data *in vivo*, EZH2i could reduce the H3K27me3 status by approximately 50% by day 2 post activation compared to vehicle control (**Figure 4B**). By day 6 post-activation, EZH2i were found to significantly reduce the viability of activated cells by nearly 30%, which only further declined over time (**Figure 4C**). Given the increased BIM observed in the scRNA-Seq data (**Figure 3E**) and donor cells (**Figure 3F**) we stained for BIM levels *in vitro.* We found significant induction compared to vehicle-treated control T cells on day 4 (**Figure S6C**), and day 6 post-activation (**Figure 4D**). To see if these findings translate to humans, we activated isolated human CD8^+^ T cells *in vitro* in the presence of EZH2i. We found a similar, potent reduction in H3K27 trimethylation status compared to vehicle control-treated T cells (**Figure 4E**) with a concomitant reduction in viability (**Figure 4F**). We also observed higher intracellular staining for BIM, consistent with the observation made in murine CD8^+^ T cells (**Figure 4D**), namely, that EZH2i exposure induces increased expression of this pro-apoptotic protein (**Figure 4G**). Together, these data support the conclusion that persistent high-dose EZH2i impairs CD8^+^ effector T cell responses leading to increased BIM expression that may contribute to the loss of CD8^+^ T cell survival in a T cell-intrinsic manner.

## Conclusions

Here, we show that using EZH2i can compromise the efficacy of α4-1BB using *in vivo* syngeneic tumor models and link this to the loss of cytotoxic CD8^+^ T cell populations. We concluded that EZH2i impair the antigen-specific CD8^+^ T cell response in terms of expansion and effector function and demonstrated these effects to be dose-dependent. Despite the compromised effector program, the generation of CD8^+^ T cell memory remains intact. Our use of scRNA-Seq provided insight into the reduced effector programming mediated by EZH2i treatment. Analysis of DEG due to EZH2i revealed an increase in BIM levels that potentially influences CD8^+^ T cell survival, which was reproduced *in vitro* using mouse and human culture systems.

Successful combination of EZH2i and IO therapies remains possible, as evidenced by previous studies that successfully promoted tumor regression (7, 9, 10). However, our studies demonstrate that EZH2i compromise the efficacy of agonistic α4-1BB, and thus not all IO agents may be candidates for combination therapy. This lack of synergy is likely because α4-1BB induces the rapid proliferation of CD8^+^ T cells requiring EZH2 for full effector differentiation and programming. These results are supported by acute viral infection models where ablation of *Ezh2* compromised the antigen-specific T cell response and recapitulated many of the phenotypes we observed here, including skewed differentiation and reduced survival (15, 16). These data emphasize the complexity of combination therapies and careful consideration that must be given when simultaneously targeting cancer and immune cells to promote robust antitumoral responses.

## Data Availability Statement

The data presented in the study are deposited in the Gene Expression Omnibus repository, accession number GSE188473.

## Ethics Statement

All experiments and procedures were conducted under approved protocols by the Institutional Animal Care and Use Committee (IACUC) (LAJ-2019-01347).

## Author Contributions

Project conception: SS-A and SP. Experiments: CJS, EH, HZ, CL, SP, and CD. Analysis: CJS, WY, EH, TX, SP, GDT, and SS-A. Manuscript drafting: CJS, GT, and SS-A. Directly provided contributions, read, and approved the final manuscript: all authors.

## Funding

Funding for this work was provided by Worldwide Research, and Development and Medical group of Pfizer Inc.

## Conflict of Interest

All authors are/were employed by Pfizer Inc. (CS, GT, EH, CD, and WY are currently employed by Pfizer Inc. SS-A, SP, and HZ were employed by Pfizer Inc.).

This study received funding from Worldwide Research, and Development and Medical group of Pfizer Inc. The funders held no part in the study design, data collection, and analysis, decision to publish, or preparation of the manuscript. All authors declare no other competing interests.

## Publisher’s Note

All claims expressed in this article are solely those of the authors and do not necessarily represent those of their affiliated organizations, or those of the publisher, the editors and the reviewers. Any product that may be evaluated in this article, or claim that may be made by its manufacturer, is not guaranteed or endorsed by the publisher.

## Abbreviations

CBI, Checkpoint blockade immunotherapy; DEG, Differentially expressed genes; EZH2, Enhancer of Zeste Homolog 2; EZH2i, EZH2 inhibitors; GSVA, Gene set variation analysis; H3K27, Histone 3 lysine 27; H3K27me3, H3K27 trimethylation; IO, Immuno-oncology; MFI, Median Fluorescence Intensity; MPEC, Memory precursor effector cell; mpk, mg/kg; NES, Normalized enrichment score; PRC2, Polycomb Repressive Complex 2; SLEC, Short lived effector cell; TME, Tumor microenvironment.
