# Supplementary material for: EZH2 Inhibition Compromises α4-1BB-Mediated Antitumor Efficacy by Reducing the Survival and Effector Programming of CD8+ T Cells
Source: Front Immunol. 2021 Nov 24;12:770080. doi: 10.3389/fimmu.2021.770080 (PMC8683156; doi:10.3389/fimmu.2021.770080)
Supplement: Supplementary file 1 [file DataSheet_1.pdf]

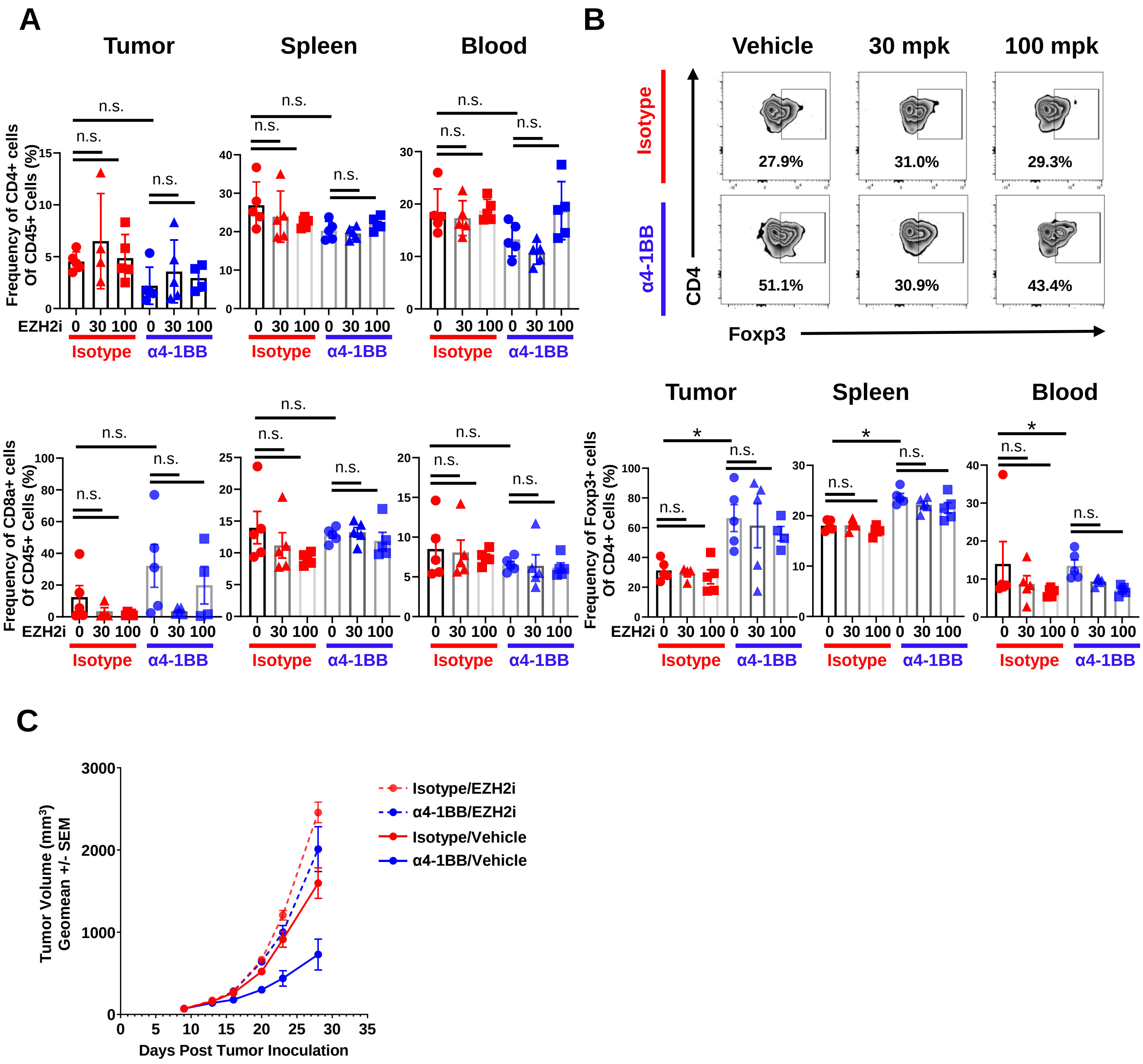

Figure S1. Differences in the frequency of T cell populations from CT26-tumor bearing mice (from Fig 1) were determined in terms of total $CD4^+$ (A, upper panel) and $CD8^+$ (A, lower panel) T cells as well as Treg cells ($CD4^+Foxp3^+$) (B), shown in representative FACs plots for tumor samples (B, upper panel), and pooled data (B, lower panel) from day 17 post implantation. C57BL/6 mice were implanted with MC38 cells and treated daily with EZH2i (at 100 mpk). When tumors reached ~70 mm^3, α4-1BB or isotype (3 mpk) was administered every 3 days for a total of 3 doses, s.c. Tumor growth rate was tracked until humane endpoint (C). For A and B, mice treated with isotype are marked in red; mice treated with α4-1BB are marked in blue. Vehicle-treated control mice are marked with circles; triangles indicate 30 mpk EZH2i treatment while squares indicate 100 mpk EZH2i treatment. n≥ 4 mice per group for CT26 tumor model (A-B). n≥ 7 mice per group for MC38 tumor studies (C). Asterisks (*) indicated significant findings; $p \leq 0.05$. n.s. indicates non-significant result.

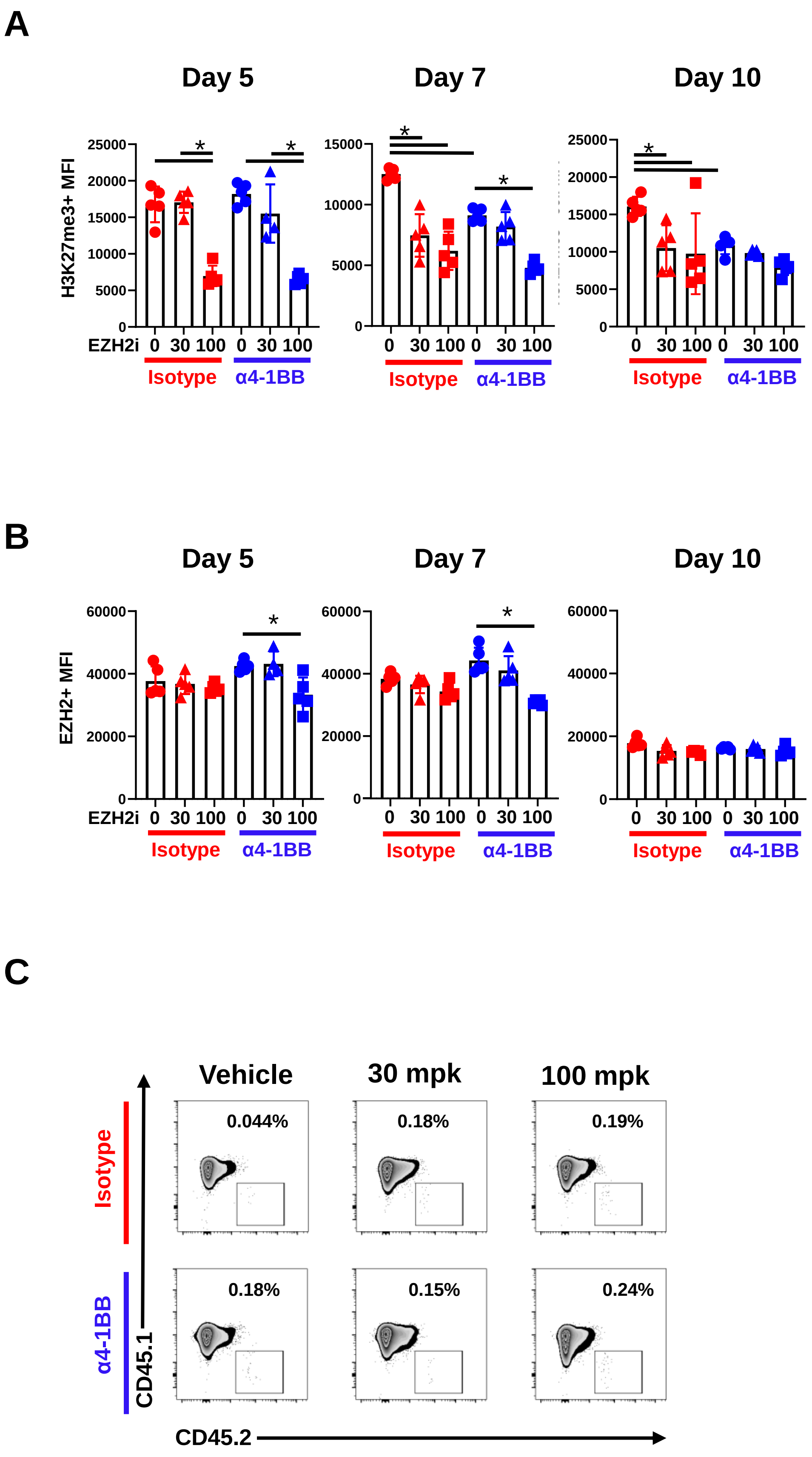

(Figure S2 continued on following page)

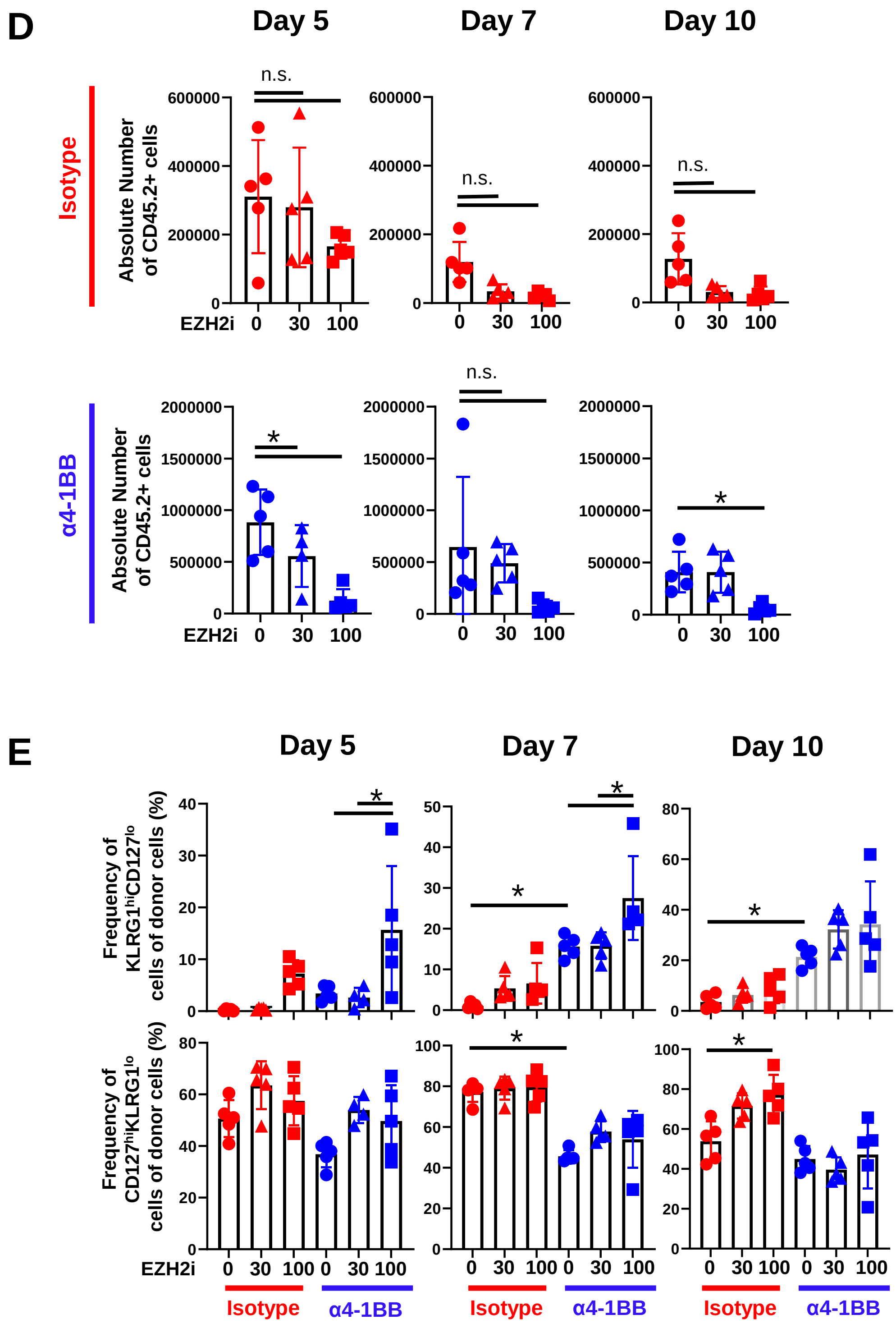

Figure S2. On days 5, 7, and 10 post immunization with OVA, donor cells were detected in recipient mice and stained for H3K27 trimethylation status (H3K27me3). The H3K27me3 (A) and EZH2 (B) median fluorescence intensity (MFI) was determined via intranuclear flow cytometry staining. Flow cytometry panels depicting frequency of endogenous (CD45.1+) versus donor (CD45.2+) activated (CD44+ pregate) splenic CD8+ T cells at day 3 post immunization (C). Absolute number of donor cells recovered from recipient animals over the course of the acute response was calculated (D). The frequency of SLEC (KLRG1^{hi}CD127^{lo}) (E, upper panel) and MPEC (CD127^{hi}KLRG1^{lo}) (E, lower panel) phenotype cells was determined by flow cytometric staining on days 5, 7, and 10 post activation. Mice treated with isotype are marked in red; mice treated with α4-1BB are marked in blue. Vehicle-treated control mice are marked with circles; triangles indicate 30 mpk EZH2i treatment while squares indicate 100 mpk EZH2i treatment. Asterisks (*) indicate $p<0.05$ as determined by 2way ANOVA and post hoc comparison of group means. n≥ 4 mice per group for immunization studies (A-E).

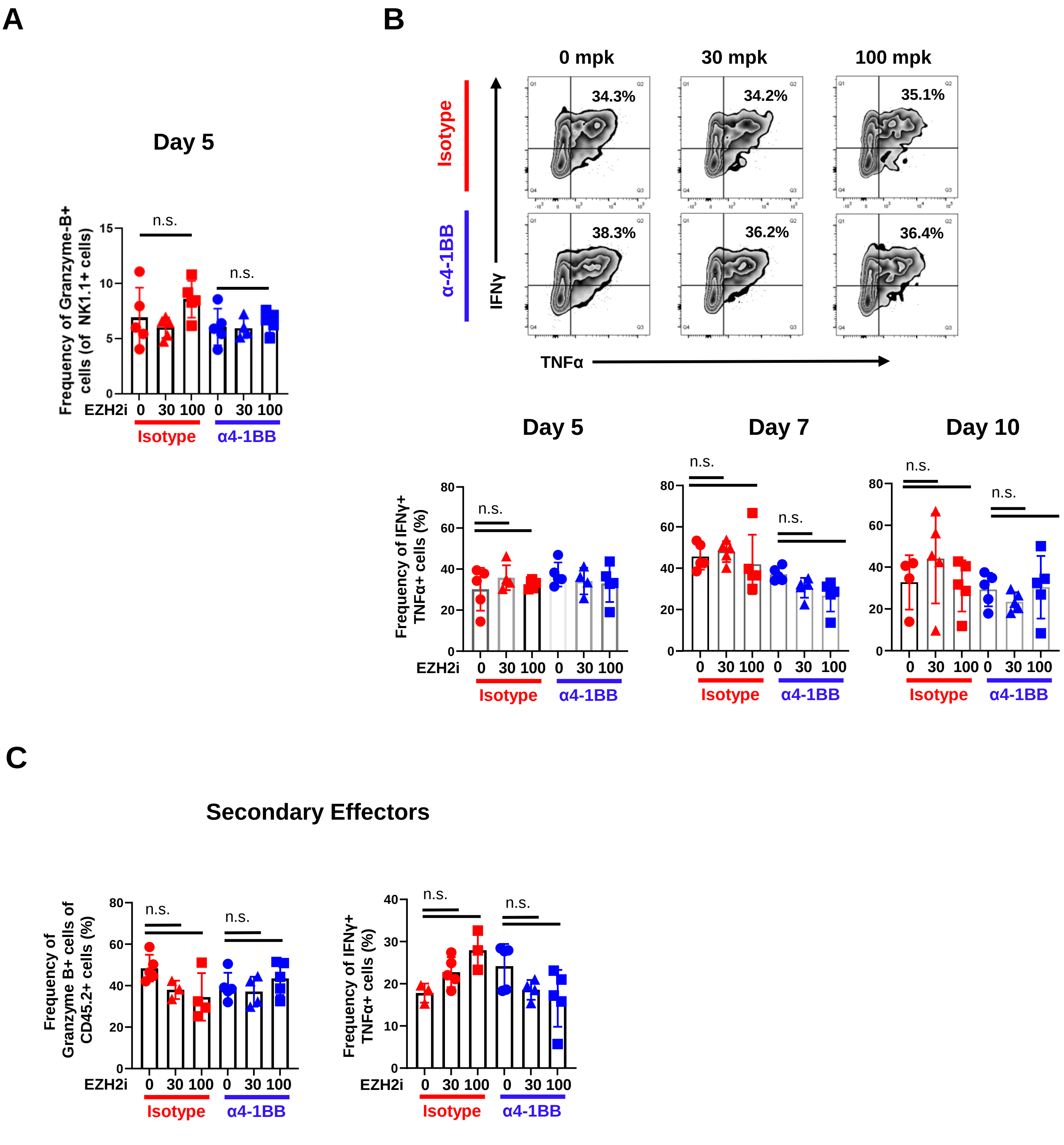

Figure S3. The frequency of Granzyme B^{+} Natural Killer cells (A) was determined by flow cytometry from mice treated with EZH2i on day 5 post OVA immunization (Fig 2). The polyfunctionality (TNFα^{+}IFNγ^{+}) of OT-I^{+} donor cells was determined after *ex vivo* stimulation with cognate antigen and intracellular staining. Representative FACs plots from day 5 stimulated samples are shown (B, upper panel) as well as bar charts showing pooled, cumulative data (B, lower panel). On day 5 post secondary OVA immunization (Fig 2E), the frequency of Granzyme B^{+} secondary effectors was determined by intracellular staining (C). Mice treated with isotype are marked in red; mice treated with α4-1BB are marked in blue. Vehicle-treated control mice are marked with circles; triangles indicate 30 mpk EZH2i treatment while squares indicate 100 mpk EZH2i treatment. Asterisks (*) indicate p<0.05 as determined by 2way ANOVA and post hoc comparison of group means. n≥ 3 mice per group for immunization studies (A-C).

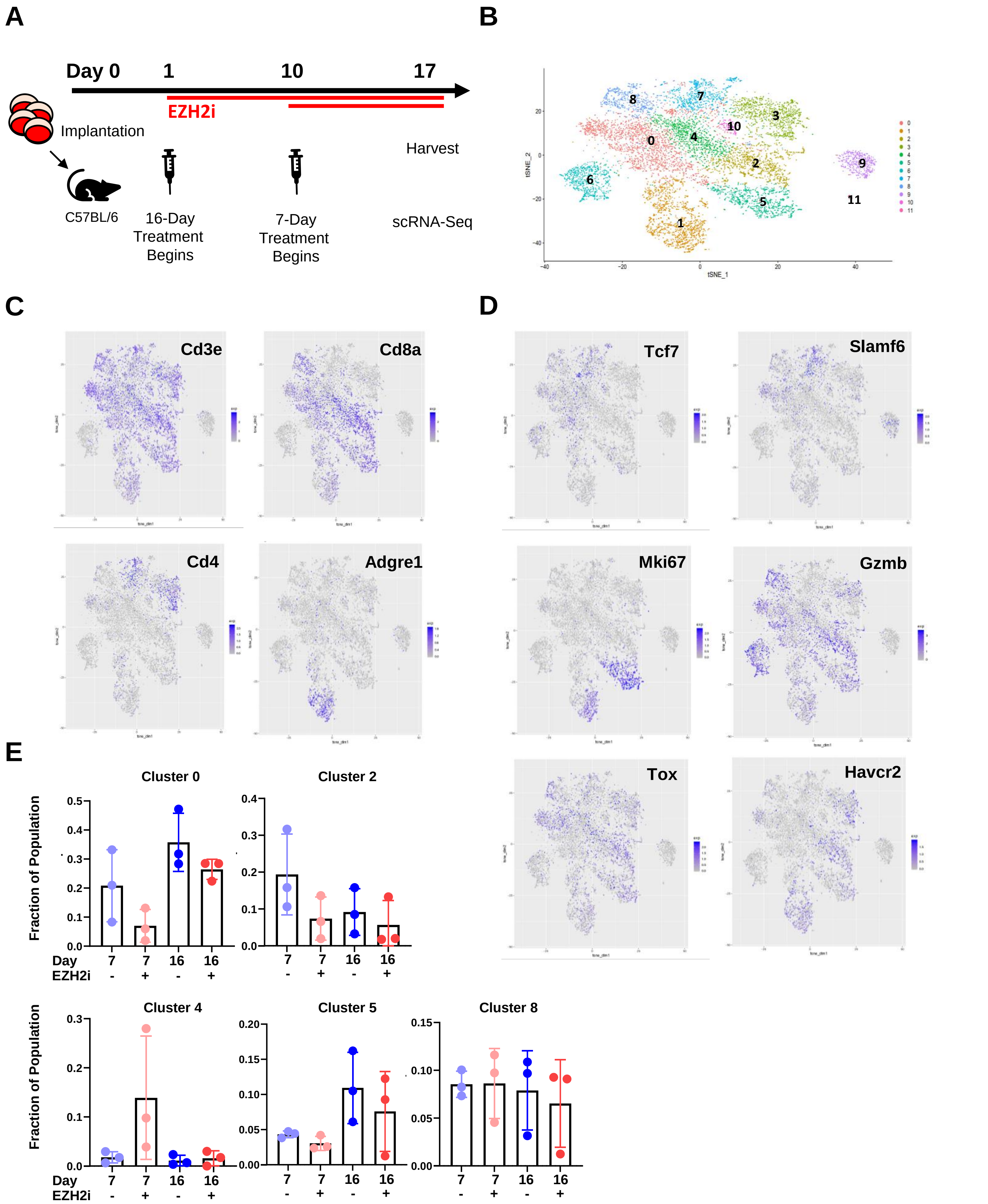

Figure S4. MC38 tumor cells were implanted in naïve mice and treated for 16 or 7 days prior to takedown with 100 mpk EZH2i (A). CD45^{+} T cells were isolated and scRNA-Seq performed with subsequent lymphocyte reclustering identifying 12 clusters (B). Feature maps depict CD8^{+} T cell clusters by high coexpression *CD3e* and *Cd8a* and low expression of *Cd4* and *Adgre1* (F4/80) (C). Features plots showing T cell cluster defining genes *Tcf7, Mki67, Tox, Slamf6, Gzmb,* and *Havcr2* (D). Dot plots are shown illustrating the fraction of each CD8^{+} cluster defined as it appears in Figure 3A after 7 or 16 days of either vehicle or EZH2i prior to isolation of CD45^{+} cells (E).

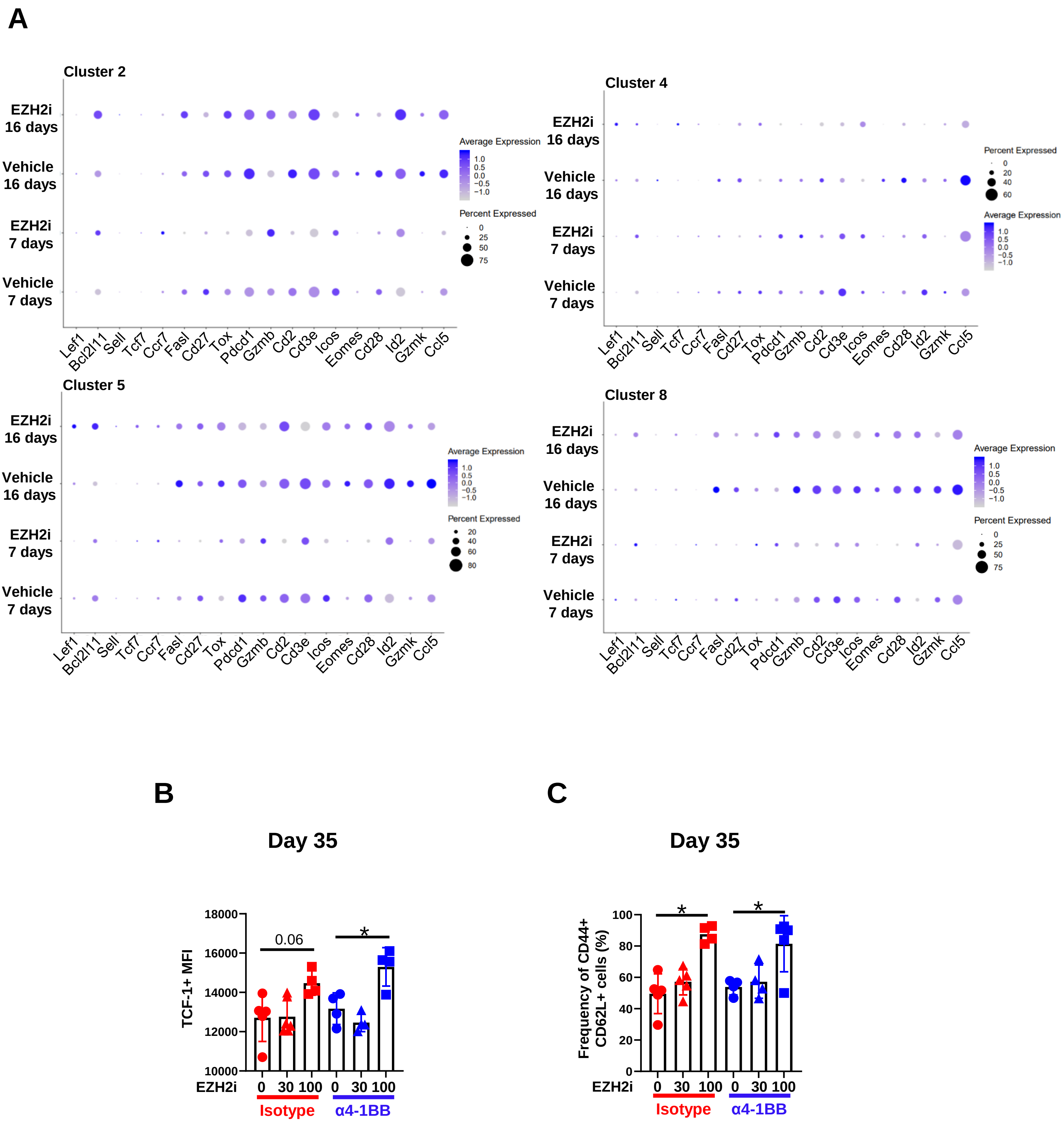

Figure S5. Curated genes of interest are depicted in dotplots from the reclustered lymphocyte CD8$^+$ T cell clusters in Figure 3A (A). From mice treated as in Figure 2E without a secondary immunization, the phenotype of resting memory cells was characterized by flow cytometric staining for expression of TCF-1 by MFI (B) and central memory phenotype (CD44$^+$CD62L$^+$) (C). Mice treated with isotype are marked in red; mice treated with α4-1BB are marked in blue. Vehicle treated control mice are marked with circles; triangles indicate 30 mpk EZH2i treatment while squares indicated 100 mpk EZH2i treatment. Asterisks (*) indicate $p<0.05$ as determined by 2way ANOVA and post hoc comparison of group means. n≥ 3 mice per group for immunization studies (B-C).

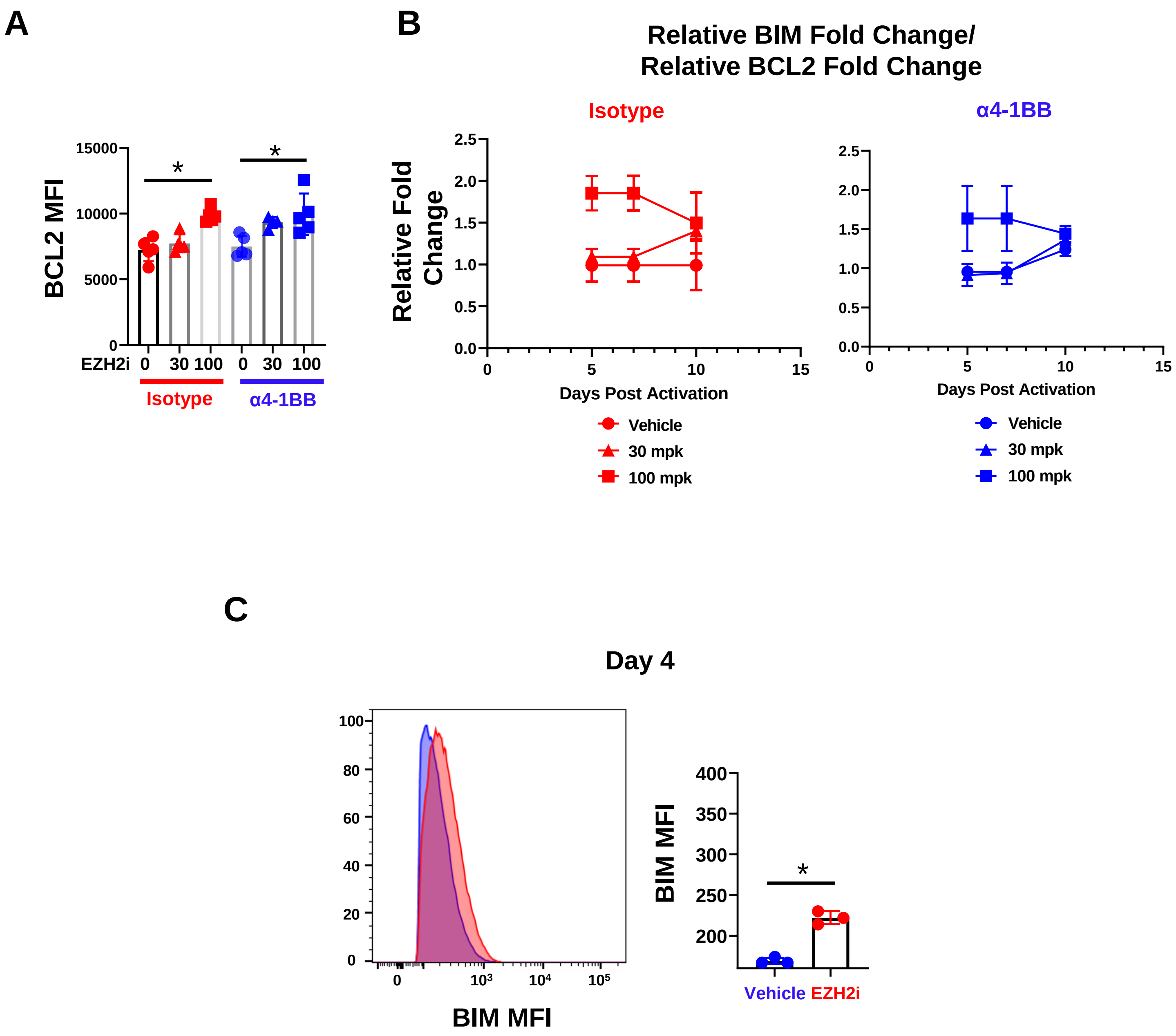

Figure S6. The induction of anti-apoptotic protein BCL2 was determined in effectors (Fig 3F) (A, left panel). The relative induction of pro-apoptotic BIM was compared to the relative induction of anti-apoptotic BCL2 and tracked over the acute response (B). I*n vitro* isolated and activated $CD8^+$ T cells were stained for BIM at day 4 post activation, shown is a representative histogram (C, left panel) and pooled data (C, right panel) of BIM MFI. For A and B, mice treated with isotype are marked in red; mice treated with α4-1BB are marked in blue. Vehicle-treated control mice are marked with circles; triangles indicate 30 mpk EZH2i treatment while squares indicate 100 mpk EZH2i treatment. Asterisks (*) indicate $p<0.05$ as determined by 2way ANOVA and post hoc comparison of group means. n≥ 3 mice per group for immunization studies. For C, blue indicates vehicle-treated control cells and red indicates EZH2i-treated cells. Gaussian distribution was determined by normality test and subsequent appropriate t-test, unpaired student's t-test or Mann-Whitney U, was performed to determine significance. Asterisks (*) indicated significant findings; $p \leq 0.05$. Results representative of 2 independent experiments.

Surface Targets	Marker	Format	Isotype	Company	Catalogue	Clone
	CD4	BUV737	Rat IgG2a, κ	BD Biosciences	612843	RM4-5
	CD4	BUV496	Rat IgG2b, κ	BD Biosciences	612952	RM4-5
	CD8a	BUV395	Mouse IgG1, κ	BD Biosciences	563795	RPA-T8
	CD8a	BUV395	Rat IgG2a, κ	BD Biosciences	563786	53-6.7
	CD8a	AF700	Rat IgG2a, κ	Biolegend	100730	53-6.7
	CD44	BUV737	Rat IgG2b, κ	BD Biosciences	612799	IM7
	CD44	BB700	Rat IgG2b, κ	BD Biosciences	566507	IM7
	CD45	PerCP	Rat IgG2b, κ	Biolegend	103130	30-F11
	CD45.1	PerCP	Mouse (A.SW) IgG2a, κ	Biolegend	110726	A20
	CD45.2	BV650	Mouse (SJL) IgG2a, κ	Biolegend	109836	104
	CD62L	PE-Dazzle	Rat IgG2a, κ	Biolegend	104448	MEL-14
	CD62L	BUV563	Rat IgG2a, κ	BD Bioscience	741230	MEL-14
	CD127	BV605	Rat IgG2a, κ	Biolegend	135041	A7R34
	NK1.1	BUV805	Rat IgG2a, κ	BD Bioscience	741993	29A1.4
	KLRG1	BV510	Syrian Hamster IgG	Biolegend	138421	MAFA, 2F1-Ag
	KLRG1	BV605	Syrian Hamster	Biolegend	138419	MAFA, 2F1-Ag
	TCRbeta	BV510	Armenian Hamster IgG2, λ1	BD Biosciences	563221	H57-597

Intracellular Targets	Marker	Format	Isotype	Company	Catalogue	Clone
	TCF-1	PE	Mouse IgG1, κ	BD Biosciences	564217	S33-966
	Tbet	BV421	Mouse IgG1, κ	Biolegend	644816	4B10
	FoxP3	AF532	Rat IgG2a, κ	eBioscience	58-5773-82	FJK-16s
	TOX	eFl660	Rat IgG2a, κ	eBioscience	50-6502-82	TXRX10
	Eomes	PE-eFl610	Rat IgG2a, κ	eBioscience	61-4875-82	Dan11mag
	H3K27me3	AF488	Rabbit IgG	CST	5499S	C36B11
	Ki-67	BUV395	Mouse IgG1, κ	BD Biosciences	564071	B56
	BIM	PE	Rabbit mAb	CST	12186S	C34C5
	Bcl-2	PE-Cy7	Mouse IgG1, κ	Biolegend	633512	BCL/10C4
	TNFa	PE	Rat IgG1, κ	Biolegend	506306	MP6-XT22
	IFNg	BV650	Rat IgG1, κ	Biolegend	505832	XMG1.2
	Gran B	Pac Blue	Mouse IgG1, κ	Biolegend	515408	GB11

Viability	Format	Company	Catalogue
	BD Fixable Viability Stain 780	BD Biosciences	565388
	LIVE/DEAD Fixable Blue Dead Cell Stain	Invitrogen (Molecular Probes)	L23105

Table S1. Antibodies and Reagents used for flow cytometric staining are listed.

	Pathway	p-value	NES
1	HALLMARK_APOPTOSIS	0.001793	1.824282653
2	HALLMARK_WNT_BETA_CATENIN_SIGNALING	0.013091	1.60330205
3	HALLMARK_KRAS_SIGNALING_UP	0.018515	1.330213665
4	HALLMARK_INFLAMMATORY_RESPONSE	0.021504	1.63793221
5	HALLMARK_MITOTIC_SPINDLE	0.025975	1.615810478
6	HALLMARK_INTERFERON_ALPHA_RESPONSE	0.036782	1.486394609
7	HALLMARK_IL2_STAT5_SIGNALING	0.036782	1.486394609
8	HALLMARK_INTERFERON_GAMMA_RESPONSE	0.04825	1.541703975
9	HALLMARK_ESTROGEN_RESPONSE_EARLY	0.055524	1.304876262
10	HALLMARK_ESTROGEN_RESPONSE_LATE	0.055524	1.304876262
11	HALLMARK_EPITHELIAL_MESENCHYMAL_TRANSITION	0.055524	1.304876262
12	HALLMARK_XENOBIOTIC_METABOLISM	0.055524	1.304876262
13	HALLMARK_ADIPOGENESIS	0.075437	1.279538859
14	HALLMARK_MTORC1_SIGNALING	0.075437	1.279538859
15	HALLMARK_OXIDATIVE_PHOSPHORYLATION	0.094685	-1.279451132
16	HALLMARK_IL6_JAK_STAT3_SIGNALING	0.120483	1.336085042
17	HALLMARK_COMPLEMENT	0.13258	1.254201456
18	HALLMARK_UNFOLDED_PROTEIN_RESPONSE	0.133897	-1.254115466
19	HALLMARK_KRAS_SIGNALING_DN	0.171026	-1.2287798
20	HALLMARK_MYC_TARGETS_V1	0.205782	-1.236089069
21	HALLMARK_UV_RESPONSE_UP	0.226472	1.190857948
22	HALLMARK_SPERMATOGENESIS	0.264349	-1.165440635
23	HALLMARK_BILE_ACID_METABOLISM	0.283114	-1.152772803
24	HALLMARK_APICAL_JUNCTION	0.394581	-1.076765805
25	HALLMARK_COAGULATION	0.468819	-1.026094473
26	HALLMARK_TGF_BETA_SIGNALING	0.521005	-0.961476944
27	HALLMARK_MYC_TARGETS_V2	0.540431	-0.944307713
28	HALLMARK_HYPOXIA	0.643017	0.876404501
29	HALLMARK_P53_PATHWAY	0.651435	-0.858461557
30	HALLMARK_ALLOGRAFT_REJECTION	0.696163	0.815783144
31	HALLMARK_TNFA_SIGNALING_VIA_NFKB	0.758971	0.776290491
32	HALLMARK_REACTIVE_OXIGEN_SPECIES_PATHWAY	0.846077	-0.772737813
33	HALLMARK_DNA_REPAIR	0.942758	0.709447288

Table S2. Complete list of Hallmark gene set database pathways upregulated with EZH2i after 16 days of treatment from scRNA-Seq data of Cluster 0 (Fig 3). Pathway name, p-value, and normalized enrichment score (NES) are given.

	Pathway	p-value	NES
1	HALLMARK_TNFA_SIGNALING_VIA_NFKB	0.004087241	-1.941503742
2	HALLMARK_ALLOGRAFT_REJECTION	0.004731227	-1.926210863
3	HALLMARK_XENOBIOTIC_METABOLISM	0.048608969	-1.532611243
4	HALLMARK_COMPLEMENT	0.057856969	-1.552105291
5	HALLMARK_INTERFERON_GAMMA_RESPONSE	0.105208612	-1.432609931
6	HALLMARK_UNFOLDED_PROTEIN_RESPONSE	0.109655815	1.264912317
7	HALLMARK_TGF_BETA_SIGNALING	0.122595769	-1.26083175
8	HALLMARK_APICAL_JUNCTION	0.135532591	1.36043346
9	HALLMARK_MYC_TARGETS_V1	0.155051816	1.240303128
10	HALLMARK_BILE_ACID_METABOLISM	0.159460651	-1.23620613
11	HALLMARK_EPITHELIAL_MESENCHYMAL_TRANSITION	0.161490683	-1.317533659
12	HALLMARK_KRAS_SIGNALING_UP	0.181067068	-1.299536811
13	HALLMARK_COAGULATION	0.229632048	-1.244392212
14	HALLMARK_FATTY_ACID_METABOLISM	0.230985134	-1.239738961
15	HALLMARK_ANDROGEN_RESPONSE	0.24824545	-1.219123633
16	HALLMARK_KRAS_SIGNALING_DN	0.290790204	-1.162966318
17	HALLMARK_ANGIOGENESIS	0.300208367	-1.142628773
18	HALLMARK_PI3K_AKT_MTOR_SIGNALING	0.317923074	-1.145825434
19	HALLMARK_INFLAMMATORY_RESPONSE	0.407636181	-1.045648527
20	HALLMARK_HEME_METABOLISM	0.422969188	1.011134174
21	HALLMARK_G2M_CHECKPOINT	0.451397444	-1.007561452
22	HALLMARK_PEROXISOME	0.467491454	-1.005380284
23	HALLMARK_HYPOXIA	0.481968397	-0.984175369
24	HALLMARK_UV_RESPONSE_DN	0.485691418	-0.97834093
25	HALLMARK_SPERMATOGENESIS	0.492143381	1.013898589
26	HALLMARK_ESTROGEN_RESPONSE_LATE	0.517284372	-0.952186533
27	HALLMARK_APOPTOSIS	0.549185113	-0.922894736
28	HALLMARK_IL6_JAK_STAT3_SIGNALING	0.633756539	-0.858241711
29	HALLMARK_OXIDATIVE_PHOSPHORYLATION	0.653941147	0.849013255
30	HALLMARK_IL2_STAT5_SIGNALING	0.667891024	-0.830696494
31	HALLMARK_DNA_REPAIR	0.675764869	-0.822911358
32	HALLMARK_ESTROGEN_RESPONSE_EARLY	0.677577313	0.828514691
33	HALLMARK_P53_PATHWAY	0.679064825	-0.817311437
34	HALLMARK_MITOTIC_SPINDLE	0.706323228	0.802798173
35	HALLMARK_CHOLESTEROL_HOMEOSTASIS	0.710055885	0.805178107
36	HALLMARK_INTERFERON_ALPHA_RESPONSE	0.711053794	-0.789888962
37	HALLMARK_ADIPOGENESIS	0.771614742	-0.746840747
38	HALLMARK_GLYCOLYSIS	0.823901932	-0.688615363
39	HALLMARK_PROTEIN_SECRETION	0.919977419	0.62524232
40	HALLMARK_MYC_TARGETS_V2	0.92777288	-0.719068107
41	HALLMARK_E2F_TARGETS	0.931159367	-0.586378868
42	HALLMARK_MTORC1_SIGNALING	0.934936405	-0.566045632
43	HALLMARK_REACTIVE_OXIGEN_SPECIES_PATHWAY	0.949592815	0.573587094
44	HALLMARK_MYOGENESIS	0.958735747	-0.561570325
45	HALLMARK_UV_RESPONSE_UP	0.992463787	0.487201711

Table S3. Complete list of Hallmark gene set database pathways downregulated with EZH2i after 16 days of treatment from scRNA-Seq data of Cluster 0 (Fig 3). Pathway name, p-value, and normalized enrichment score (NES) are given.
